# Give more data, awareness and control to individual citizens, and they will help COVID-19 containment

**DOI:** 10.1007/s10676-020-09572-w

**Published:** 2021-02-02

**Authors:** Mirco Nanni, Gennady Andrienko, Albert-László Barabási, Chiara Boldrini, Francesco Bonchi, Ciro Cattuto, Francesca Chiaromonte, Giovanni Comandé, Marco Conti, Mark Coté, Frank Dignum, Virginia Dignum, Josep Domingo-Ferrer, Paolo Ferragina, Fosca Giannotti, Riccardo Guidotti, Dirk Helbing, Kimmo Kaski, Janos Kertesz, Sune Lehmann, Bruno Lepri, Paul Lukowicz, Stan Matwin, David Megías Jiménez, Anna Monreale, Katharina Morik, Nuria Oliver, Andrea Passarella, Andrea Passerini, Dino Pedreschi, Alex Pentland, Fabio Pianesi, Francesca Pratesi, Salvatore Rinzivillo, Salvatore Ruggieri, Arno Siebes, Vicenc Torra, Roberto Trasarti, Jeroen van den Hoven, Alessandro Vespignani

**Affiliations:** 1 ISTI-CNR, Pisa, Italy; 2 IAIS-Fraunhofer, Sankt Augustin, Germany; 3 City University of London, London, UK; 4 Northeastern University, Boston, USA; 5 IIT-CNR, Pisa, Italy; 6 ISI Foundation, Turin, Italy; 7 Eurecat, Barcelona, Spain; 8 University of Torino, Turin, Italy; 9 Sant’Anna School of Advanced Studies Pisa, Pisa, Italy; 10 Penn State University, State College, USA; 11 King’s College London, London, UK; 12 Umeå University, Umeå, Sweden; 13 Universitat Rovira i Vir-gili, Tarragona, Catalonia Spain; 14 University of Pisa, Pisa, Italy; 15 ETH Zurich, Zurich, Switzerland; 16 Aalto University School of Science, Espoo, Finland; 17 Central European University, Budapest, Hungary; 18 Technical University of Denmark, Lyngby, Denmark; 19 FBK, Trento, Italy; 20 DFKI, Kaiserslautern, Germany; 21 Dalhousie University, Halifax, Canada; 22 Polish Academy of Sciences, Warsaw, Poland; 23 Universitat Oberta de Catalunya, Barcelona, Spain; 24 TU Dortmund University, Dortmund, Germany; 25 ELLIS Alicante, Alicante, Spain; 26 Data-Pop Alliance, New York, USA; 27 Universita degli Studi di Trento, Trento, Italy; 28 MIT, Cambridge, USA; 29 EIT Digital, Povo, Italy; 30 Universiteit Utrecht, Utrecht, The Netherlands; 31 Maynooth University, Maynooth, Ireland; 32 TU Delft, Delft, The Netherlands

**Keywords:** COVID-19, Personal data store, Mobility data analysis, Contact tracing

## Abstract

The rapid dynamics of COVID-19 calls for quick and effective tracking of virus transmission chains and early detection of outbreaks, especially in the “phase 2” of the pandemic, when lockdown and other restriction measures are progressively withdrawn, in order to avoid or minimize contagion resurgence. For this purpose, contact-tracing apps are being proposed for large scale adoption by many countries. A centralized approach, where data sensed by the app are all sent to a nation-wide server, raises concerns about citizens’ privacy and needlessly strong digital surveillance, thus alerting us to the need to minimize personal data collection and avoiding location tracking. We advocate the conceptual advantage of a decentralized approach, where both contact and location data are collected exclusively in individual citizens’ “personal data stores”, to be shared separately and selectively (e.g., with a backend system, but possibly also with other citizens), voluntarily, only when the citizen has tested positive for COVID-19, and with a privacy preserving level of granularity. This approach better protects the personal sphere of citizens and affords multiple benefits: it allows for detailed information gathering for infected people in a privacy-preserving fashion; and, in turn this enables both contact tracing, and, the early detection of outbreak hotspots on more finely-granulated geographic scale. The decentralized approach is also scalable to large populations, in that only the data of positive patients need be handled at a central level. Our recommendation is two-fold. First to extend existing decentralized architectures with a light touch, in order to manage the collection of location data locally on the device, and allow the user to share spatio-temporal aggregates—if and when they want and for specific aims—with health authorities, for instance. Second, we favour a longer-term pursuit of realizing a Personal Data Store vision, giving users the opportunity to contribute to collective good in the measure they want, enhancing self-awareness, and cultivating collective efforts for rebuilding society.

## Introduction

National authorities are currently addressing the rapid spread of SARS-CoV-2 through strong control measures aimed at containing the diffusion of the virus and slowing it to levels that can be managed by health-care and socio-political institutions. Knowing where and when the diffusion is taking place is essential for shortening the emergency period, and for better focusing countermeasures where they are actually needed. The imposition of generalized and strong emergency measures limiting citizen liberty appears to be not the optimal approach, and it is, in part, an effect of our poor knowledge of how and where exactly the virus is circulating and outbreaks are growing.

Personal big data, in particular those able to describe the movement of people in greater detail, should be seen as a potentially powerful weapon in combatting the pandemic. For example in contact tracing, that is, revealing the places a patient who has tested positive has visited in recent days (thus identifying places in risk of contagion) in addition to the people the user has been in contact with (thus identifying specific people at risk). Indeed, very recent simulation results (Ferretti et al. 2020) have clearly shown that immediate tracing of infected people—ideally from the pre-symptomatic phase – could significantly contribute to reducing the individual’s infection rate (the well-known R0 index) below 1. Also, it has been found that over 75% of individuals who reported being positive for COVID-19 had been in close contact with another individual—who they knew—infected by COVID-19 (Oliver et al. 2020).

Analyzing contact traces and other individual mobility data potentially raises risks for the individual privacy, and that is at the core of current debates about the best trade-off between privacy and data value for public health. One example is South Korea, which made the movement of positive patients de facto public, clearly favouring data value (with excellent results in containment of virus spread) while sacrificing patient privacy (who risks a social stigma, potentially dissuading people from exposing and testing for the virus Zastrow 2020). In contrast, various European efforts lean toward strong individual personal data protection, stipulating clear requirements that data collection apps should satisfy (10 requirements for the evaluation of “Contact Tracing” apps 2020) and promoting a unified European approach (Manancourt 2020). We believe it is possible to reap the benefits of contact tracing, including collection of location data with a privacy preserving level of granularity, without forgoing personal data protection altogether while enhancing trust. GDPR compliance, abiding to the principle of privacy/data protection by design and privacy/data protection by default, enables the benefits of location data.

## Existing proposals and their limitations

Learning from current success stories as well as controversies, various teams of researchers and developers are now proposing a different vision where privacy protection is a must, and solutions are designed to extract useful data without sharing personal sensitive information. In particular, the spatial information associated with the individual citizens (where they stay or move) is considered to be too sensitive, and difficult to protect. An important research direction is the privacy-safe, spatially-oblivious implementation of proximity-tracing, that in this context basically represents the ability to reconstruct the close contacts with other people that an individual had before being tested positive. A strongly decentralized representative of this direction is the DP3T (Decentralized Privacy-Preserving Proximity Tracing 2020) approach. The solution is based on mobile phone apps that continuously collect the list of anonymous, dynamically changing, app-generated IDs of other phones (which, therefore, need to have the app installed, too) that had close and prolonged contacts with the device. With DP3T, the trusted authority simply broadcasts the anonymous app-generated IDs of the positive patient’s phone, and each contact needs to check the list to find themselves. The recent joint effort by Apple and Google to provide Android and iOS system-level support for contact tracing through and hoc APIs (Apple and Google Partner on COVID-19 Contact Tracing Technology 2020) also goes in this direction. A similar view, yet leaning towards a centralized management of the anonymous contact traces, is provided by the PEPP-PT initiative (Pan-European Privacy-Preserving Proximity Tracing 2020), where the broadcasting phase is replaced with a different communication way where positive users provide the list of “contacted” anonymous app-generated IDs to the trusted central authority, who is then able to directly call and warn the phones in the list^1^.

The strong point of these approaches lies in the simplicity of the information used, which allow easy and rapid implementations able to guarantee privacy protection (obviously stronger in the completely decentralized solutions). While we believe that these approaches are on the right track and particularly useful in the short term, we also emphasize that limiting the analysis to simple contact (close-range proximity) data limits the efficacy. One point is that the discoverability of potentially exposed contacts is by design limited to those who have the app installed (both the positive person and the exposed one), making it impactful only after a critical mass of users is reached (some models suggest 60% is the optimal threshold (Digital Contact Tracing Can Slow or Even Stop Coronavirus Transmission and Ease Us out of Lockdown 2020)). Also, only direct contacts are detected, thus not considering surface-touch contamination, which is a typical phenomenon in large shared spaces, like supermarkets and such, considered to be a potential vector of diffusion (van Doremalen 2020). Another important task would be to quickly detect outbreak hotspots, and for this purpose spatial and temporal information could be a key ingredient. Spatial-temporal information within a privacy preserving architecture (e.g. appropriate granularity levels, clear access rights and aims for data processing, enhanced security, etc.) can provide vital granular aggregate data with a modest or null impact on fundamental rights and freedoms, see for example the MIT Private Kit Safe Path initiative (Raskar et al. 2020).

## Our proposal

Our claim is very simple: limiting data flow at its very source is not the best answer. Individual citizens—and only them—should be able to collect detailed information about their own position and movement, together with other types of data, including (in the direction of previous proposals) pseudo-IDs of devices at close distance. The means for safeguarding privacy should instead be in providing the users with full control of such data, together with the necessary tools for sharing only the information they want at the preferred level of detail, to customise sharing of information depending on the individuals/entities with whom they are sharing, and for evaluating pros and cons of each sharing option.

The paradigm we envision is based on a Personal Data Store (Giannotti et al. 2012; de Montjoye et al. 2014; Study xxxx) where users collect and manage all their own data, equipped with data management and analytics tools for elaborating them, as well as with functionalities for controlling what kind of information—raw or derived from data – should be shared with other users or with authorities. The main points of the approach we envision, based on such environment, are the following:

1. Each user has a personal software environment (either directly on the smart phone or in the cloud) where they can store, elaborate and control their own data in an exclusive way. No third-party has access to this data.
2. The personal software environment of the user is a tool they can actively decide to use to perform actions, for instance to help in providing correct information to health authorities in case they are tested positive to COVID-19; or simply to contribute to public safety by joining some collective computation of global statistics useful to improve countermeasures. We stress the fact that any sharing of the user’s data or aggregates must happen only if the user wants to, and no authority should be able to access anything without the user’s consent. The PDS aims to empower the individual’s memory and inference capabilities, and its inviolability should be guaranteed both by technological means and by its social recognition as a basic right of individuals.
3. When the user decides to share information, they define the aggregates to share, taking into consideration the minimum spatial and temporal granularity of the information needed to realize the service. The environment provides the functionalities to define the minimum data requirement and to compute and share the data. A key point is that deciding the best trade-off between privacy and data utility might require a knowledge that is available only late in the process, because the context might change either the utility of a given type of data or the priorities of the individual.
4. The information sharing can happen in two modalities:
  A simple transfer to a trusted authority of the minimum data needed to realize the service, for instance the list of close contacts (e.g. as hashed mac-addresses that only the contact themselves can recognize) similar to PEPP-PT and DP3T; or the list of locations visited, in the form of Points of Interest or municipalities, useful to find potential outbreaks hotspots.
  When possible, through a collaborative, distributed computation of global aggregates involving the information of the user, e.g. using secure multi-party computation techniques Lindell and Pinkas (2008) or specific privacy-preserving distributed methods (Meng-Chang 2012).
5. The data shared by users during and for the COVID-19 emergency must be treated in accordance with two basic requirements: Use Limitation Principle and finite, contextual life-span of data, i.e. the data collected is used solely for the purpose of COVID-19 containment, and the life-span is limited and declared at the time of collection, and the data will auto-destruct at the end of the period. Both constraints can only partially be implemented by means of policies, and technical tools must be developed to verify that the policies on use are actually followed and implemented, such as adopting formal methods of program verification or cryptographic data auto-destruction techniques—both still not adequately developed to scale as needed. Finally, standards for personal mobility and proximity data management, defining policies regulating data gathering, storage and destruction need to be developed by joint bodies that will include researchers from the government, industry and academia.
6. The information provided by authorities (possibly thanks to the individual contributions) about risky areas and possible contacts with positive patients can be joined with the complete information the user has about themselves, providing a data analytics-enabled self-awareness of own behaviour and the potential points of risk. For instance, the user might not realize that their own daily home-work routine involves passing through an area that has an increased level of risk, and the analysis might suggest modifying the route to work. Note that such level of detailed information would only be available locally to the specific user, as it comes from “merging” global information provided by centralised entities (e.g., a nation-wide authority) with local information available only to the individual user (on their devices).

This proposal (like the DP3T and PEPP-PT initiatives) exemplifies a distinctively European approach of aiming to co-realize important moral obligations—in this case for public health and saving lives, together with respecting rights and fundamental freedoms – instead of choosing for a quick solution that relativizes one of our conflicting obligations.

Maintaining the trust of citizens at a time of crisis like the current one is a priority. This includes respecting the requirements for maintaining fundamental respect for human rights, ethical principles and existing legislation. A user-centric approach will also ensure that data is only used during the duration of the crisis and that the user has the control to end the tracking once the need is over. Our proposal leverages both the respect for individual freedoms and for the environment, by cultivating feelings of solidarity and a sense of collective responsibility for rebuilding society.

## Recommendations

Summarizing, our view is that emergency situations like the COVID-19 pandemic represent a strong case—yet one not unique—where providing people complete control of the data they produce and collect and how they are shared (and, maybe, an improved awareness of what they are collecting) can provide an edge in facing complex challenges. Initiating (centralized or decentralized) data collection with rigid predefined privacy and data quality requirements and excluding the human from the decision loop can often be suboptimal.

Our main recommendation, therefore, is to work on two parallel tracks: in the short-term and in the long-term. In the short term, the decentralized architectures currently under development for social contact tracing (in particular, PEPP-PT and D3PT) should be extended to manage the collection of location data locally on the device. A loose integration between the two components (contact and location data) should be provided, that keeps them logically independent and mutually not linkable. This allows us to maintain all the privacy-by-design benefits of the contact tracing solutions mentioned above, but the moment users are confirmed as a positive case, it allows them to voluntarily provide additional contextual information in a privacy-preserving way (on top of independently triggering the PEPP-PT or D3PT contact tracing mechanisms), that can contribute to the computation of useful global aggregates (European Commission 2020), such as spatio-temporal density maps (Monreale et al. 2013) to identify potential infection hubs through location data.

Over a longer-term, deep-impact actions should be investigated to realize a Personal Data Store approach, where the user can collect all their data in a decentralized, safe and controlled way, equipped with tools to analyze the data and understand its potential value (for instance, to contribute improving public good) as well as the potential consequences that sharing data or aggregates can have on their privacy. The aim is to enable more effective emergency countermeasures based on a novel connection between collective good and the huge information treasure that each individual brings with themselves.

## Footnotes

1 Due to its centralized perspective and to alleged transparency issues, the PEPP-PT model has been later at the center of discussions, various key partners left the consortium and its weight in the European scenario shrank significantly. At the time of writing, the reference website and GitHub repository have not been updated for several months.

## References

[CR6] Apple and Google partner on COVID-19 contact tracing technology (2020). Apple.com newsroom, 10 April 2020. https://www.apple.com/newsroom/2020/04/apple-and-google-partner-on-covid-19-contact-tracing-technology/

[CR3] 10 requirements for the evaluation of “Contact Tracing” apps (2020). Chaos Computer Club. https://www.ccc.de/en/updates/2020/contact-tracing-requirements

[CR12] de Montjoye Y-A, Shmueli E, Wang SS, Pentland AS (2014). openPDS: protecting the privacy of metadata through SafeAnswers. PLoS One.

[CR8] Digital contact tracing can slow or even stop coronavirus transmission and ease us out of lockdown (2020). Big Data Institute, Univ. of Oxford. https://www.bdi.ox.ac.uk/news/digital-contact-tracing-can-slow-or-even-stop-coronavirus-transmission-and-ease-us-out-of-lockdown

[CR5] DP3T: decentralized privacy-preserving proximity tracing (2020). https://github.com/DP-3T/documents

[CR17] European Commission (2020). COMMISSION RECOMMENDATION of 8.4.2020 on a common Union toolbox for the use of technology and data to combat and exit from the COVID-19 crisis, in particular concerning mobile applications and the use of anonymised mobility data. https://ec.europa.eu/info/sites/info/files/recommendation_on_apps_for_contact_tracing_4.pdf

[CR1] Ferretti, L., Wymant, C., Kendall, M., Zhao, L., Nurtay, A., Bonsall, D.G., & Fraser, C. (2020). Quantifying dynamics of SARS-CoV-2 transmission suggests that epidemic control is feasible through instantaneous digital contact tracing. Science. https://science.sciencemag.org/content/early/2020/03/30/science.abb6936 10.1126/science.abb6936 PMC7164555 32234805

[CR11] Giannotti F, Pedreschi D, Pentland A, Lukowicz P, Kossmann D, Crowley J, Helbing D (2012). A planetary nervous system for social mining and collective awareness. European Physical Journal: Special Topics.

[CR15] Lindell, Y., & Pinkas, B. (2008). Secure Multiparty Computation for Privacy-Preserving Data Mining. IACR Cryptology ePrint Archive. 2008. 197. 10.29012/jpc.v1i1.566.

[CR4] Manancourt, V. (2020) EU data regulator calls for pan-European COVID-19 app. https://www.politico.eu/article/coronavirus-europe-data-regulator-calls-for-pan-european-covid-19-app/

[CR16] Meng-Chang Liu (2012). Achieving privacy-preserving distributed statistical computation. PhD Thesis, University of Manchester, UK. https://www.escholar.manchester.ac.uk/uk-ac-man-scw:166980

[CR18] Monreale Anna, Wang Wendy Hui, Pratesi Francesca, Rinzivillo Salvatore, Pedreschi Dino, Andrienko Gennady L, Andrienko Natalia V (2013). Privacy-preserving distributed movement data aggregation. AGILE Conference.

[CR14] Oliver, N, Barber, X., Roomp, K. & Roomp, K., (2020), “The Covid19Impact Survey: Assessing the Pulse of the COVID-19 Pandemic in Spain via 24 questions”, arxiv:2004.01014

[CR7] Pan-European privacy-preserving proximity tracing (2020). https://www.pepp-pt.org/

[CR10] Raskar, R., et al. (2020). Apps gone rogue: Maintaining personal privacy in an epidemic. arXiv preprint arXiv:2003.08567

[CR13] Study on Personal Data Stores conducted at the Cambridge University Judge Business School. https://ec.europa.eu/digital-single-market/en/news/study-personal-data-stores-conducted-cambridge-university-judge-business-school

[CR9] van Doremalen N (2020). Aerosol and surface stability of HCoV-19 (SARS-CoV-2) compared to SARS-CoV-1. The New England Journal of Medicine.

[CR2] Zastrow, M. (2020) South Korea is reporting intimate details of COVID-19 cases: has it helped? Nature. https://www.nature.com/articles/d41586-020-00740-y 10.1038/d41586-020-00740-y 32203363

